# Reliability of Using Texture Analysis of Periapical Radiographs Obtained Using Radiovisiography for Assessing Bone Quality in Dental Implant Planning: A Cross-Sectional Study

**DOI:** 10.7759/cureus.32860

**Published:** 2022-12-23

**Authors:** Kunchala Sailasri, Sri Guru Mangala Deepti Ganji, Parth Satwalekar, Sandeep Nalla, Ram B Basany, Seelam Sai Krishna

**Affiliations:** 1 Department of Prosthodontics and Crown and Bridge, Sri Venkata Sai (SVS) Institute of Dental Sciences, Mahabubnagar, IND; 2 Department of Prosthodontics and Crown and Bridge, Practitioner in Dr. Motiwala Dental Clinic and Implant center, Hyderabad, IND; 3 Department of Prosthodontics and Crown and Bridge, Practitioner in Satwalekar’s Multispeciality Dental Hospital, Hyderabad, IND

**Keywords:** radiovisiography, implant planning, anatomical location, texture analysis, bone quality, reliability, intra oral radiographs

## Abstract

Background

Pre-prosthetic implant radiographic imaging helps in the quantitative and qualitative analysis of the bone structure and also enables the evaluation of the relationship between critical structures and potential implant sites.

Purpose

The aim of the study was to define the reliability of utilizing the analysis of textures from the periapical radiographic images obtained through Radiovisiography (RVG) in order to better plan for dental implantations.

Methods

A cross-sectional study was conducted using 50 intraoral periapical radiographs which were obtained through RVG performed prior to the placement of implants. The radiographs were segregated based on anatomical locations i.e. 12 for the anterior maxilla, nine for the posterior maxilla, seven for the anterior mandible, and 22 for the posterior mandible. Each of the radiographs was visually assessed by four experienced examiners, namely a Prosthodontist E1, Periodontist E2, Oral surgeon E3, and Oral radiologist E4, which was then compared to an experienced operator's tactile perception during a pilot drill for implant placement. As a reference, the Lekholm and Zarb classification was provided to all the examiners for them to qualitatively assess the bone structure in the radiographs.

Results

The examiners’ results were correlated with the assessment provided by the experienced operator. E1 and E4 successfully assessed 42% of the radiographs while E2 had the least success with only 26%. Of the 12 anterior maxillary radiographs, only eight were accurately assessed by E1. With respect to the posterior maxilla, all examiners correctly assessed four radiographs each. Of the seven anterior mandibular radiographs, except for E2, the rest correctly assessed three radiographs each. Of the 22 posterior mandibular radiographs, only nine were accurately assessed by E4.

Conclusion

Intraoral periapical radiographs obtained through RVG did not meet the desired parameters for assessing the bone quality during the planning stage for implants.

## Introduction

The greatest contribution of computer technology in health sciences, including dentistry, is the advent of radiographic image-based diagnoses and interpretation [1,2]. Computer-aided diagnosis (CAD) is referred to as the process by which diagnosis is provided through the automation of quantitative image analysis, thereby improving the accuracy of diagnosis and assisting in clinical decisions [3]. Using technology, texture analysis detects spatial variances by utilizing the differing intensities of grey levels, which are obtained through the local pixel variations that are repeated along with the image either regularly or randomly. There are many techniques for texture analysis that are grouped into one of four viz. structural, statistical, fractal and anisotropy [4]. Haralick et al. also defined texture analysis as being 2-dimensional wherein in the first dimension, the grey levels (pixels) are arranged primitively in relation to one another, and in the second dimension, their spatial relationships are defined [4]. Texture analysis has often been used for evaluating the density of bone tissues in patients with osteoporosis while the fractal analysis method is more commonly used in dentistry for evaluating jawbone sites [5-7]. Analysis of fractal dimensions has been exhibited to predict bone qualities surrounding dental implants [8], and has been tested on the alveolar bone surrounding dental implants after loading of prosthesis [9].

The success of dental implantation is dependent on the quantity and quality of the jawbone. In order to better plan for dental implants, the characteristics of jaw bones have been measured using a combination of pre and trans-operative methodologies [10]. For ensuring successful implantation, the stability of primary implants has been shown to be a significant factor [11]. Quantitative or subjective determination of the characteristics of bone morphologies before any procedure could potentially aid in the prediction of the stability of implants and their successful osseointegration. Quantitative determination of bone can be achieved through bone mapping or by Cone-beam computed tomography (CBCT). Qualitative analyses have been performed by utilizing panoramic and periapical radiographs coupled with other advanced diagnostic procedures such as Dual Energy X-Ray Absorptiometry (DEXA), Computed tomography (CT), and CBCT [12]. However, CT scans have a few disadvantages, such as increased radiation dosages, costs, and the fact that not every patient may need one for planning dental implants [13]. If the alveolar ridge buccolingual width is adequate, an intraoral radiograph might be sufficient for assessment of the implant site to check whether it is situated farther from sensitive anatomies [14]. In dentistry, intraoral periapical (PA) radiographs are routinely performed and widely available. Usage of these radiographs for the assessment of bone quality would result in qualitative analysis of the bone and its patterns, cost reduction during implantation, and reduced exposure to radiation typically encountered in CT scans [14].

The objective of this study arose from the lack of availability of CBCT in every clinic or dental office, which may force implant practitioners to rely on intra-oral periapical radiographs for quantitative and qualitative implant planning. Therefore, a feasibility study was performed to observe the usefulness of periapical radiographs obtained using RVG without the help of adjunct methods. The cross-sectional study investigated the possibility of utilizing periapical radiographs obtained using RVG during the planning process prior to dental implantation.

## Materials and methods

**Figure 1:**
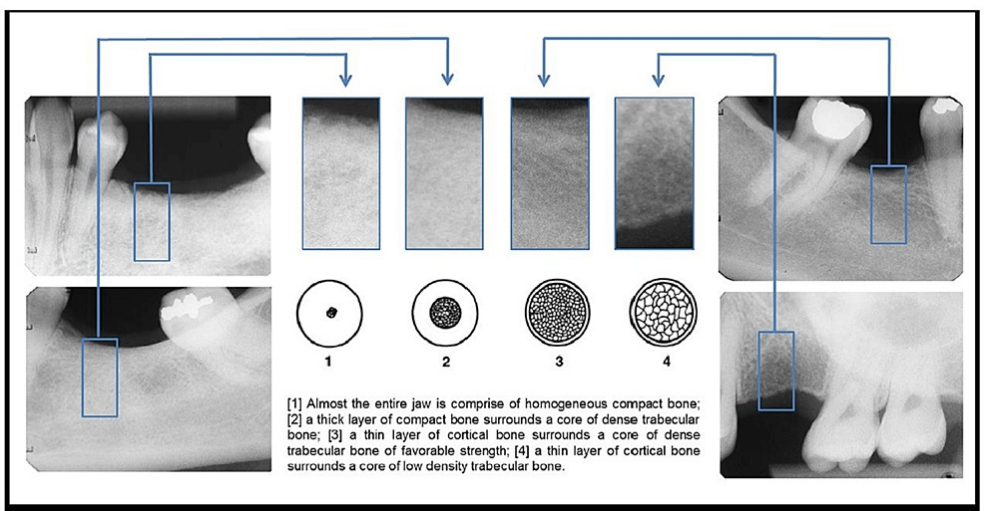
Jawbone site radiographic images according to Lekholm and Zarb’s bone classification

This cross-sectional study was done at Sri Venkata Sai (SVS) Institute of Dental Sciences, Mahabubnagar, in compliance with the ethical standards description of the Helsinki Declaration and the United States Federal Policy for Protection of Human Subjects. The study protocol was approved by the SVS Institute of Dental Sciences Institutional Ethics Committee with ethical approval number Svsids/Prosth/1/2019. The study also adhered to the Strengthening the Reporting of Observational studies in Epidemiology (STROBE) guidelines for cross-section studies [15]. A pilot analysis was performed for assessing the suitability and for estimating the number of samples needed. The pilot analysis indicated that a sample of 49 radiographs would be required at a ratio of var1/var0 = 1.75, at a power of 90%, and α = 0.05. Therefore, 50 intraoral periapical radiographs of 50 different patients (N=50) were obtained using Radiovisiography. The respondents chosen were those with an indication of dental implant treatment, with good oral hygiene, and of the age of 18 years and above and willing to give consent. Respondents were rejected if they presented poor oral hygiene and had local/systemic conditions contraindicated for implant surgical procedures. The obtained radiographs using RVG were segregated based on their anatomical locations which resulted in 12 for the anterior maxilla, nine for the posterior maxilla, seven for the anterior mandible, and 22 for the posterior mandible. The periapical radiographs were performed using an RVG 5100 System, KODAK Carestream Dental, USA. Standard parameters were 65kVp, and 10mA, and exposure time was 0.3 secs for posterior teeth and 0.2 secs for anterior teeth. The captured images were saved in JPEG format. The visual examination of these radiographs for bone quality evaluations was individually assessed by four experienced examiners viz. a Prosthodontist E1, Periodontist E2, Oral surgeon E3, and Oral radiologist E4. All examiners were successful practitioners and designated as heads of the department in the institution at which the current research study was performed. E1, E2, and E3 were selected since these specialists performed implant procedures and E4 was selected, as the person was experienced in radiographic interpretation. According to the classification of Lekhom and Zarb, the bone quality [16], is divided into 1, 2, 3, and 4 as illustrated in (Figure 1).

**Figure 2:**
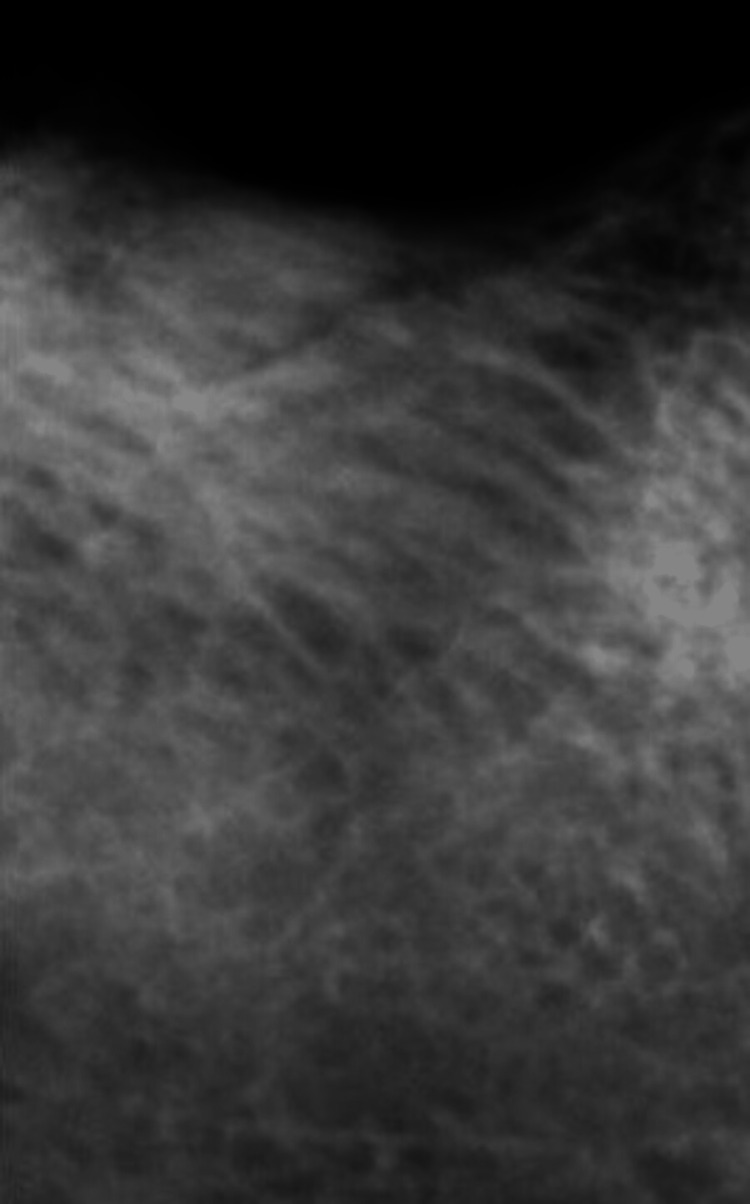
Sample radiographic image of a respondent presented to the examiners for bone quality type analysis.

The image containing this classification was presented to all the examiners to act as a reference when evaluating the bone quality of the given radiographic image. The obtained radiographic images from the respondents were cropped and rotated in such a way that only the edentulous portion was visualized without any surrounding teeth and anatomical structures, following which it was presented to each examiner individually for a qualitative analysis of the bone type as illustrated in (Figure 2).

This was done to remove any bias that may aid in the identification of the anatomical location. On the other hand, for these same jaw bone sites, an experienced operator (age 48), practicing from the year 1997, and performing an average of 60 implants per year, registered the bone quality based on the aforementioned classification, by using tactile perception during a pilot drill for implant placement. The results provided by each examiner were then tabulated for the final evaluation. A two-step assessment of the bone type was performed. Initially, interpretations of the radiographs were done based on the image of an edentulous portion, which was cropped and rotated without any surrounding teeth and anatomical structures. This was further matched to an image with a schematic bone type drawing using the Lekholm and Zarb classification as a reference, following which the bone type was noted. During surgery, the experienced surgeon used their tactile perception to assess the bone resistance and registered the bone type, after performing the first drilling. The data obtained was stored on Microsoft Excel worksheets for further statistical analyses. The Chi-square test was performed and results were tabulated.

## Results

The Chi-square test (Table 1) revealed that there were no significant differences between the assessments of the four specialists (p < 0.05) when evaluating the bone type.

**Table 1:** Frequency distribution of study radiographs in evaluating the type of bone by four specialty groups

Assessment of examiners correlated to operator		Prosthodontist (E1)	Periodontist (E2)	Oral surgery (E3)	Oral radiologist (E4)	Total	P value
Yes	Count	21	13	14	21	69	.170
	% within Groups	42.0%	26.0%	28.0%	42.0%	34.5%	
	% of Total	10.5%	6.5%	7.0%	10.5%	34.5%	
No	Count	29	37	36	29	131	
	% within Groups	58.0%	74.0%	72.0%	58.0%	65.5%	
	% of Total	14.5%	18.5%	18.0%	14.5%	65.5%	
Note: p value less than 0.05 was considered stastistically significant							

The results also indicated (Table 2) that during the qualitative analysis of the bone type, there were no significant differences when evaluating the type of bone quality at different anatomical locations.

**Table 2:** Frequency distribution of study radiographs in evaluating the type of bone at different anatomical locations

Assessment of the examiners correlated to the operator		Anterior maxilla	Posterior maxilla	Anterior mandible	Posterior mandible	Total	P value
Yes	Count	8	4	3	9	24	.524
	% within Groups	66.7%	44.4%	42.9%	40.9%	48.0%	
	% of Total	16.0%	8.0%	6.0%	18.0%	48.0%	
No	Count	4	5	4	13	26	
	% within Groups	33.3%	55.6%	57.1%	59.1%	52.0%	
	% of Total	8.0%	10.0%	8.0%	26.0%	52.0%	
Total	Count	12	9	7	22	50	
Note: p-value less than 0.05 was considered statistically significant							

Table 3 highlights the characteristics of the respondents in this study.

**Table 3:** Respondent's characteristics(N=50)

Variable	Characteristics	Frequency (N= 50)	Percent
Age	18 - 40	15	30
	41 – 60	25	50
	61-80	10	20
Sex	Male	29	58
	Female	21	42
Bone quality type noted by operator	Type 1	5	10
	Type 2	20	40
	Type 3	20	40
	Type 4	5	10
Accurate evaluation of bone quality type by examiners correlated to operator	E1	21	42
	E2	13	26
	E3	14	28
	E4	21	42
Accurate evaluation of bone quality type at different anatomical locations	Anterior maxilla	8 out of 12	66.7
	Posterior maxilla	4 out of 9	44.4
	Anterior mandible	3 out of 7	42.9
	Posterior mandible	9 out of 22	40.9

**Figure 3:**
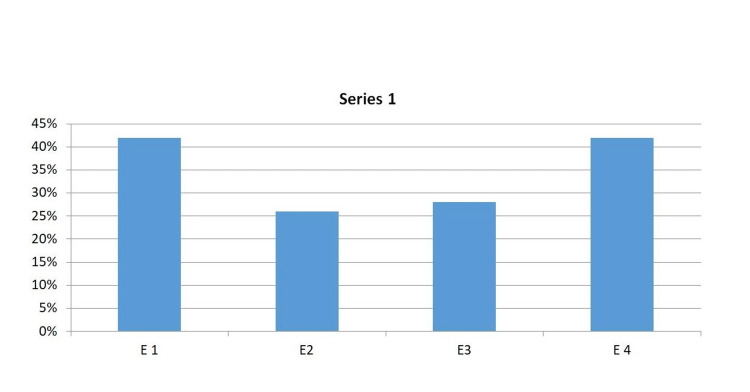
Examination accuracy percentage based on operators' tactile perception

Of the total of 50 implant sites, 21 were situated in the maxilla and 29 in the mandible. The results from the examiners were in direct correlation to the assessment of the operator’s tactile perception as seen in (Figure 3). E1 and E4 obtained 42% success in assessing the radiographs, followed by E3 at 28% and the least being E2 at 26%.

**Figure 4:**
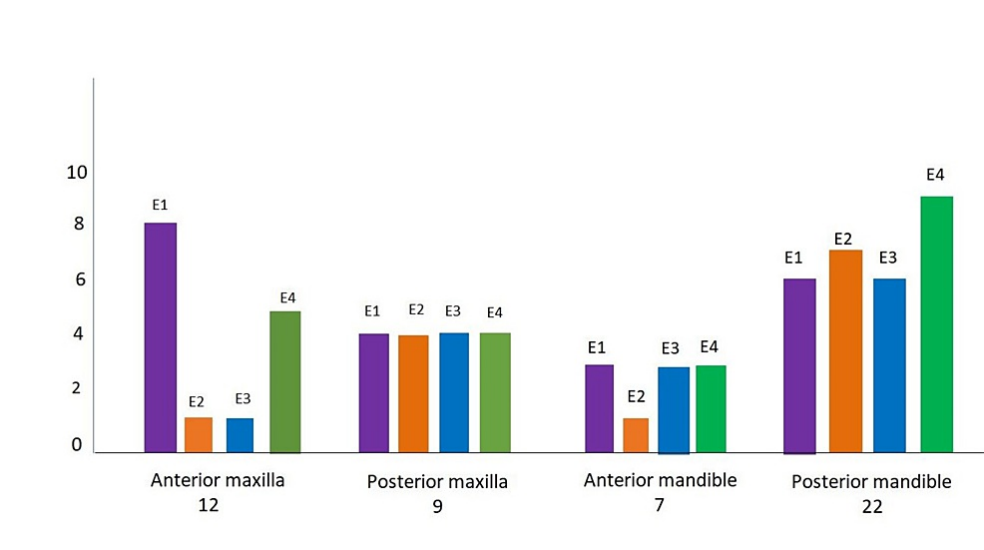
Results of the examiners according to the anatomical location

Figure 4, presents the results of the examiners according to the anatomical location. Of the 12 anterior maxillary radiographs, only eight (66.7%) were accurately assessed by E1, which was the highest among all examiners. In the posterior maxilla, out of nine radiographs, all examiners correctly assessed four (44.4%) radiographs each. Of the seven anterior mandibular radiographs, except for E2, all others correctly assessed three (42.9%) radiographs each. Of the 22 posterior mandibular radiographs, only nine (40.9%) were accurately assessed by E4, which was the highest among all examiners.

**Figure 5:**
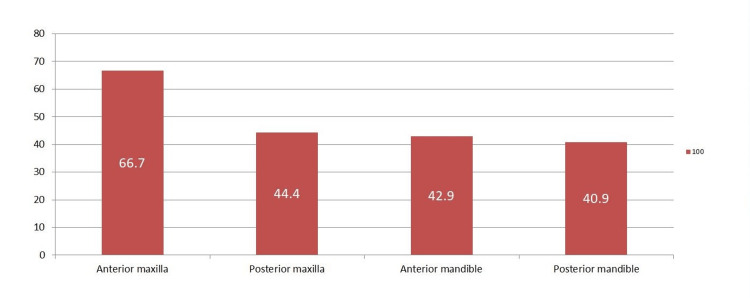
Accuracy percentage of bone quality type evaluation at different anatomic locations

Figure 5 displays the accuracy percentage of bone quality type evaluation at different anatomical locations. The highest accuracy was observed in the analysis of anterior maxilla radiographs and the least was observed in the posterior mandible radiographs. Bone type 1 showed more gray levels with less variation between them and enhanced textural homogeneity. Grey levels were fewer in type 4, with greater variation and heterogeneity between them. Intermediate characteristics were observed with respect to three attributes in bone type 2 and bone type 3, with grey tones wider in type 3 in comparison to type 2.

## Discussion

This study enhances existing literature by identifying periapical radiograph texture analysis as a means of qualitatively distinguishing the 4 bone types based on one of the existing classification methods. This study employed the classification postulated by Lekholm and Zarb (1985), mainly due to its global usage and fidelity [17]. In this classification, illustrations of the jaws are given along with text for delineating bone tissue type. The quality of bone is then grouped into one of four types based on the proportions and design of the compact and trabecular bone tissue [12]. If the tissues are comprised of homogenous cortical bone, then it is classified as Type 1. Conversely, Type 4 bone tissues typically present with thin cortical bone enclosing sparsely set low-density trabecular bone. Under the intermediate groups, if the trabecular bone is dense and is enclosed by a thick layer of cortical bone, then it is grouped under Type 2, whereas Type 3 tissues consist of dense trabecular bone surrounded by a thin layer of cortical bone.

It is to be noted that several other different classifications grouping bone tissues have also been employed in the past [17], which include the Misch Classification (1990), Trisi and Rao classification (1999), and the classification put forth by the University of California, Los Angeles (UCLA). In the Misch Classification [12], the density of the bone in the edentulous regions based on the tactile and macroscopic characteristics of the cortical and trabecular bone was used as parameters for the grouping into D1, D2, D3, and D4. If the cortical bone is dense, it is grouped in D1. If a coarse trabecular bone is enclosed by a dense and porous crest cortical bone, then is grouped under D2. If the crest cortical bone is thin and porous and encloses a fine trabecular bone, then it is grouped under D3. Finally, D4 consists of fine trabecular bone. Under the Trisi and Rao classification [18], the grouping was done by correlating hand-assessed bone quality to the histomorphometric bone density into dense, normal, and soft groups. The UCLA classification [19] also has 4 types to it based on the amount and pattern of the edentulous alveolar bone in a 3-D configuration. Under Type I, it is deemed that optimum bone is present in the height and width dimensions, making it ideal for placing implants. If the bone quantity on the buccal side is deemed to be insufficient, then it is placed under Type II. In Type III, the alveolar bone is knife-shaped or the buccal side bone is deficient in volume but has optimum height. Finally, Type IV bones have improper alveolar height and width, leading to the implants being exposed on all sides. Since there are different methods of classifying bone tissues, this may end up confusing and interfering with the comparisons with other studies. However, this study utilizes the classification of Lekholm and Zarb since it is often cited in other studies of dental implant treatment. Moreover, since the classification is based on conventional radiography and is close to the objectives of the current study, it was chosen as the classification of choice.

Among the factors for predicting the outcome of implant treatment, Bone Quality (BQ) has been noted by past studies as being a significant factor. The definition of BQ has been contentious [20] since it depends on how various objects are interpreted. These include the density of bone minerals, size of the skeleton, micro-design of the trabeculae and its 3-D orientation, vascularization, and properties of the bone’s matrices. Simultaneously, BQ has also been defined as the topographic relationship of cortical and cancellous bone when the recipient site drilling is done. Four clusters are present for the BQ assessment methods. The unusual methods cluster consists of those obtained by DEXA, Resonance frequency, Periotest, and Occlusal radiography. Perioperative methods cluster include those obtained through peak insertion torque and tactile perception. Sectional imaging cluster refers to those obtained via CBCT and computed tomography and plain films cluster refers to those obtained through periapical and panoramic radiographs.

To determine the morphology and anatomical structures of the implantation site, radiography plays a vital role during the planning process. The radiographic image data is employed in the dimension estimation of the implant, its number, determining the location of the orientation of implants as well as determining the need for augmentation of the bone structure [21]. For this, differing radiographic techniques are currently used in the planning and evaluation phase of implantation. In spite of its usefulness, panoramic and periapical radiographic images exhibit differing densities due to extraneous factors affecting the radiograph itself or the post-processing methods used. To counter this, morphological evaluations have been used to characterize bone trabecular morphologies [22]. For measuring bone densities, Quantitative Computed tomography and Dual Photon X-Ray Absorptiometries can also be used but this is restricted in usage due to equipment scarcity in clinics, procedural complexity, and higher radiation dosage [22]. White et al. [23] stated that the analysis of morphometries falling under valid diagnosed levels is not impacted by the angle of radiation or image contrast.

CT scans permit accurate three-dimensional analyses of anatomies along with direct bone density measurement, a value measured in Hounsfield units (HUs) [12]. These HUs are standard numbers that originate from CT images [24]. Off late, CBCT has begun replacing multi-slice CT in dental usage for analyzing mineralized tissues due to its image quality and low exposure to radiation dosages. However, the technique has the inherent disadvantage of not being able to display the actual HU akin to the CT images. Conversely, studies on qualitative bone assessments using fractal dimensions exist, thereby predicting implantation success by tying them to values of insertion torque [22]. However, this method is generally viewed as unfavorable since it depends on the values of grey tones in the CBCT images, the stability of which may be affected by the scanner itself. Texture Analysis (TA) has hitherto been unused in dentistry, in spite of its potential, due to complexities in the interpretation phase. TA would be beneficial if it was included as an auxiliary tool to analyze images after CBCT scans [25].

A past study by Ritter et al. [26] analyzed the bone surrounding implants in dogs by employing periapical radiographs, conical beam computed tomography, and histometry. The study noted a correlation between the periapical radiographs and histometry with regard to implant length alone while no correlation was present between bone level and thickness. However, a result of the study [27] seems to suggest that using periapical radiographs would be an acceptable procedure for evaluating the density of bone before implantation since the images were capable of detecting differences in the jaws examined, along with significant correlation with the classification of Lekholm and Zarb. Nevertheless, this study emphasizes not to use of panoramic radiographs since results are varied, doesn’t allow for detecting differences between the densities of alveolar bone in both of the jaws, and doesn’t significantly correlate to the Lekholm and Zarb Classification.

For successful osseointegration, it has been noted that primary implant stability is a key factor which in turn is related to bone quality [27]. Chong et al [28] noted a stronger correlation between the initial stability of an implant to the corresponding bone density as opposed to the implant design itself. It has also been noted that the prediction of bone quality could potentially help in predicting the optimum healing period and help in deciding the loading period of the implant. For predicting primary implant stability, subjective bone classification is used, as well as Peak insertion torque and Resonance frequency analysis (RFA). It may be concisely summarized that RFA could prove useful in the prediction of post-surgical prognosis following implantation [22].

**Figure 6:**
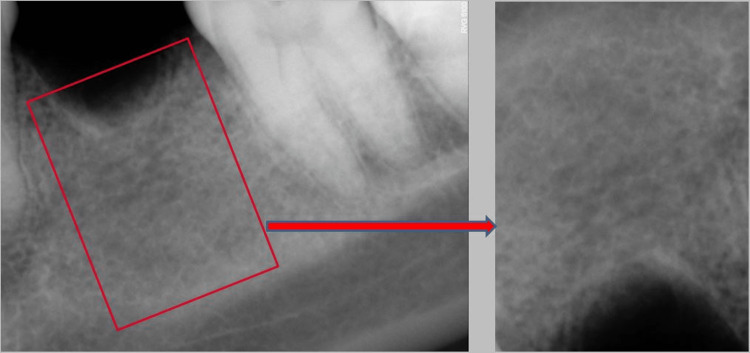
Same image has been cropped and rotated in such a way that only the edentulous portion is visualized without any surrounding teeth and anatomical structures and was given to the examiner.

In this study, the obtained radiographic images from the respondents were cropped and rotated following which it was presented to the examiner to prevent bias as exhibited in (Figure 6).

This was done to avoid the common tendency for interpreting bone quality by viewing the teeth and surrounding anatomical structures like the inferior alveolar canal, mental foramen, inferior border of the mandible, and maxillary structures like the nasopalatine canal, nasal septum, and the maxillary sinus. All these might mislead in accurately assessing bone quality. It has been noted in the past [1] that Type 1 bone is primarily cortical in nature with an opaque radiographic image. Moreover, the gradual widening of medullary spaces is observed, along with increased radiolucency in bones of type 2, 3, and 4.

The results of the present study showed that all the examiners failed to correctly interpret at least 50% of the radiographs, though there was no statistical significance between the examiners. It was also observed that the interpretation of the anterior maxilla by the examiners was relatively more accurate 66.7% followed by the posterior maxilla at 44.4%, the anterior mandible at 42.9%, and the posterior mandible at 40.9%. Since the maxilla comprises fine trabecular bone, either of D3 or D4 type, it can reasonably be assumed that the examiners could more easily analyze it due to the existence of marrow spaces. This is the opposite for the mandible which is comprised of dense and opaque cortical bone of D1 or D2 type, leading to confusion in choosing the right type. Moreover, the Lekholm and Zarb classification is based on conventional radiography and histology components but it is ambiguous as to whether the evaluation of radiographs was conducted during or preceding the surgery. It has also been noted that the classification’s accuracy level was on the lower side, especially when classifying the sparse trabeculation of the mandible [19].

This study also differs from the one conducted by Mundim et al. [1] in that the current study utilized direct digitization of images that could potentially enhance repeatability via precision error reduction which is often linked to the digitization of radiographic films [29]. It is also to be noted that another past study [30] stated that using the modified Lekholm and Zarb classification in CBCT possessed high repeatability, which was suggestive of its ability in distinguishing different combinations of cortical and cancellous bones, thereby presenting the ability to define treatment regimens and optimize results.

Limitations

This study noted that texture analysis of periapical radiographs would not be in the analysis of bone type without the presence of teeth or other anatomical structures. This may be due to one of two reasons. Firstly, the examiners may be more likely to make errors during the individual classification of each site. Another limitation of the study was its short duration and there was only one specialty-wise observer. Hence, the results must not be generalized. Also, the obtained sample sizes were unequal due to the aforementioned short duration in conjunction with the poor awareness levels among respondents since the study was performed in a remote area. Despite the study not finding significant differences between different anatomical locations, it is recommended that equal sample sizes of the anatomical locations be considered, a more extensive sample size be obtained and a longitudinal study be conducted in order to better analyze the bone quality. It is also recommended to consider the average values of multiple specialty observers rather than considering a single observer as the potentiality of the individual observer might affect the results.

One other drawback of the current study stems from the clinical interpretation of general bone quality from the entire radiographic image and not from specific areas of the image itself. Therefore, it would be beneficial to conduct further studies focusing on differentiating and evaluating bone quality at the crestal and apical level of the implant. For this study, the trans-operative tactile perception of the experienced operator was the sole method of obtaining data pertaining to implant selection, loading, and prognosis. Based on the results of the study, it can be concluded that non-invasive routine radiographical images cannot be used as a potential means of predicting bone quality.

## Conclusions

The majority of the techniques which are imaging-based have their own set of pros and cons. This presents a need for combining varied methods for optimizing diagnostic and treatment outcomes. However, two-dimensional periapical and panoramic radiographic images have the drawback of not imaging on a 3-D plane, thereby having limited use. The current study postulates that clinical professionals must choose an optimum imaging method for diagnosis rather than relying on one available at their clinic. The decision must be based on clinical examination, treatment requirements, and data derived from radiographs, thereby aiding in diagnosis.

Based on the boundaries of the current research, it can be stated that intraoral periapical radiographs alone do not meet the desired parameters in assessing bone quality. Tactile perception is an existing standard protocol that allows for the differentiation of bone types with acceptable confidence. Even though modern radiographic techniques can be used as an adjunct along with existing conventional techniques, the first step in planning dental implantation remains conventional radiographs, irrespective of whether the Lekholm and Zarb classification would be used. This is more so in cases of sufficient residual bone which negates the requirement for additional bone-based reconstruction surgical procedures.
